# Stability-Aware Geographic Routing in Energy Harvesting Wireless Sensor Networks

**DOI:** 10.3390/s16050696

**Published:** 2016-05-14

**Authors:** Tran Dinh Hieu, Le The Dung, Byung-Seo Kim

**Affiliations:** 1 Department of Electronics and Computer Engineering in Graduate School, Hongik University, Sejong 30016, Korea; trandinhhieu1989@gmail.com (T.D.H.); thedung_hcmut@yahoo.com (L.T.D.); 2 Department of Computer and Information Communication Engineering, Hongik University, Sejong 30016, Korea

**Keywords:** wireless sensor networks, energy harvesting, multi-hops routing, geographic routing

## Abstract

A new generation of wireless sensor networks that harvest energy from environmental sources such as solar, vibration, and thermoelectric to power sensor nodes is emerging to solve the problem of energy limitation. Based on the photo-voltaic model, this research proposes a stability-aware geographic routing for reliable data transmissions in energy-harvesting wireless sensor networks (EH-WSNs) to provide a reliable routes selection method and potentially achieve an unlimited network lifetime. Specifically, the influences of link quality, represented by the estimated packet reception rate, on network performance is investigated. Simulation results show that the proposed method outperforms an energy-harvesting-aware method in terms of energy consumption, the average number of hops, and the packet delivery ratio.

## 1. Introduction

Wireless sensor networks (WSNs) are widely used, although battery capacity is a major limitation of their applicability. A new class of sensors, equipped with an energy harvesting module, is a likely solution to this problem because these energy-harvesting wireless sensor networks (EH-WSNs) can provide longer network lifetimes using harvest-aware energy management [1,2]. In [3], the authors utilized solar-powered WSNs to establish a topology in which some nodes received and transmitted packets without consuming limited battery resources. While a wide variety of harvesting modalities are now feasible, solar energy harvesting through photo-voltaic conversion provides the highest power density, which makes it the best option for powering embedded systems that consume several mW through small harvesting modules [4]. Solar cells can generate a power density of 15 mW/cm^2^, compared to the 330 μW/cm^3^, 116 μW/cm^3^, 40 μW/cm^3^, and 960 nW/cm^3^ generated by piezoelectric shoe inserts, small vibration microwave ovens, thermoelectricity, and acoustic noise, respectively. Therefore, solar energy harvesting-based WSNs were adopted for the proposed protocol and performance evaluations.

Many routing protocols for EH-WSNs have been proposed. In [5,6], the authors maximized workloads that were autonomously sustained by a network, although these studies only used some of the power map patterns that represent the influence of shadowing areas on the amount of energy harvested at each node. Because the shapes of the power map patterns are very simple, this model is somewhat ideal but unrealistic. Therefore, the authors in [7] used the solar irradiation statistics database from the National Solar Radiation Database (NSRDB) to create simulations and proposed a route selection method that chooses a route based on the amount of energy consumed by transmitting packets and the harvested energy’s wastage due to overcharge. This paper only considered how to optimize the average residual energy and minimize residual energy at each node, and traffic was characterized by sequential and non-overlapping connection streams from one source to one sink. Additionally, the authors in [8] designed an adaptive opportunistic routing (AOR) protocol for EH-WSNs, which divides possible sets of intermediate nodes into different priority transmission regions, such that an intermediate node in the $k_{th}$ region will be transmitted in the $k_{th}$ time slot as long as the node has enough energy and the packet has not been relayed by higher-priority nodes. For high data rate applications using EH-WSNs, lots of duplicated data packets may be generated, although this can be overcome by using a stability-aware cooperative routing scheme for reliable high-speed data transmission in multi-rate mobile *ad-hoc* wireless networks [9].

In WSNs, connectivity requires global topology information, which can be significant in large-scaled networks and can cause increased overhead in these networks. The geographic routing method in [10,11,12,13,14] is considered an appropriate approach for large-scale WSNs because it does not require global topology information from WSNs.

Therefore, this paper proposes a geographically-based routing selection method that selects routes based on residual energy, energy harvesting, link quality, and location information to improve the performance of EH-WSNs. Actually, the performance of conventional *ad-hoc* wireless networks under the effect of imperfect link quality has been widely studied. However, to the best of our knowledge, the impact of link quality on network performance of energy harvesting wireless sensor networks still remains an open issue. The proposed Stability-Aware Geographic Routing in Energy-Harvesting wireless sensor networks (SAGREH) can be classified as geographic routing protocol like ORPL [15]. However, the method of obtaining link quality information to take it into consideration is different compared with that of ORPL. Specifically, ORPL is based on Expected Duty Cycles (EDC), a multipath equivalent of Expected Transmissions Count (ETX) [16]. The ETX metric measures link quality by calculating the expected transmissions of a packet necessary for it to be received successfully. Thus, it is considered as a passive link quality measurement because an observation time is needed. However, our proposed SAGREH can be considered as an active link quality measurement because it estimates the link quality (*i.e.*, Packet Reception Rate, PRR) from the information of the distance between two nodes by using Equation (11). Moreover, our proposed SAGREH routing protocol accumulates link quality to calculate the quality in terms of PRR of all available paths from source node to sink node. Then, it selects the most reliable path for data delivery. This investigation showed that the proposed routing method outperforms the comparative routing method in terms of packet delivery ratio, average hop count, and average energy consumption when network parameters such as radio transmission range and number of nodes vary.

The paper is organized as follows. In Section 2, the motivation for this research and related work is discussed. Section 3 introduces the system model, while Section 4 describes energy-harvesting-aware routing and the proposed method. Simulation results are shown in Section 5, and finally, the paper is concluded in Section 6.

## 2. Paper Overview

### 2.1. Related Work

Several works on energy-harvesting routing protocols have been published. In [5,6,17], the authors maximized the workload that can be autonomously sustained by a network that can transmit a packet, through an edge, proportional to the maximum flow of the edge, which was calculated using an extension of the Ford–Fulkerson algorithm. The authors in [18] developed a model to characterize the performance of multi-hop radio networks using energy constraints and design-routing algorithms to optimally utilize available energy. In [19], the authors presented an energy management and routing method that maximized energy sustainability by modeling the energy buffer as a G/G/1(/N) queue. The authors in [7] proposed a novel route selection method that considered network energy wastage due to overcharging of finite-capacity batteries, based on dynamic source routing, which is an on-demand method. The disadvantage of on-demand routing is that each node requires the full routing path in the packets forwarded from the source to destination. In a dynamic source routing protocol, a node aggressively caches all overheard source routes to reduce the propagation scope of other nodes’ flooded route requests [11]. When network nodes expand, the routing packets must establish routing paths.

**Figure 1:**
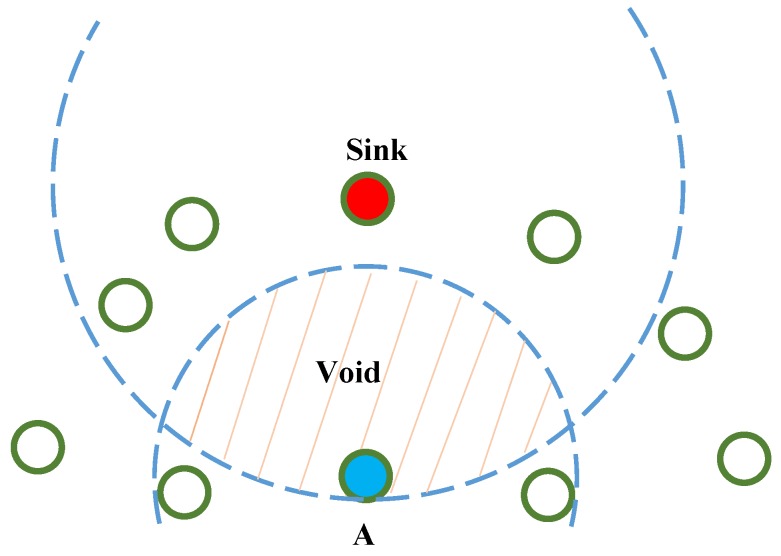
An example of local minimum problem.

Geographic routing is an appropriate approach for large-scale WSNs because it allows nodes to be nearly stateless, and each node makes routing decisions based on the location information for itself, its neighbors, and the sink node. However, geographic routing cannot optimize the number of hops when a node has no neighbor closer to the sink node, which is known as the local minimum problem. When using a greedy forwarding method, packets are stuck at node A because no neighboring node can be found closer to the sink node (Figure 1). This study, despite adopting geographic routing for EH-WSNs, attempted to solve the local minimum problem in an energy-harvesting environment by incorporating residual energy, location information, and PRR in routing path selection.

Various geographic routing protocols have been proposed. Greedy perimeter stateless routing (GPSR) is a fundamental geographic routing protocol [11] with high successful packet delivery rates, although it requires high extra costs to maintain planar graph information on sensor nodes.

In [20], the authors used geographic routing and defined energy distance as a routing metric encoded in energy information. More specifically, the distance between a sender and a receiver is transformed to a weighted distance. A node with less energy along its path is further away, *i.e.*, the energy distance. This concept finds the shortest distance path based on the minimum total energy distance, rather than physical distance. It does not take into account the influences of wireless link qualities or collisions on packet transmissions.

The authors in [8] proposed an adaptive opportunistic routing (AOR) protocol based on the geographic routing method for multi-hop EH-WSNs. The protocol achieved high throughput using a region scheme that utilized network conditions and energy availability, although in cases of high data rates, this protocol may produce too many duplicate data packets over networks.

The authors in [21] proposed a contention-based strategy of geographic forwarding for wireless sensor networks. Their first solution combines a splitting tree algorithm (random relay selection) with sectoral decision regions; this allows them to define circular sectors in order to increase the probability of finding a relay, and thus reducing the contention resolution interval. On the other hand, their second solution resorts to an auction-based relay selection and convex-lenses decision region. In this solution, the decision area is halved at every round, which allows the elimination of some candidate relays from the auction, and therefore the contention resolution interval is significantly reduced.

In [22], the authors propose a cross-layer optimized geographic node-disjoint multipath routing algorithm. The proposed routing algorithm achieved shorter average path length without causing much energy consumption, and a considerable increase of the network sleep rate was achieved.

In [23], inspired by the structure composed of edge nodes around which there is no routing void, an efficient bypassing void routing protocol based on virtual coordinates was proposed in this paper. The proposed protocol achieved higher delivery ratio, shorter path length, less control packet overhead and energy consumption.

### 2.2. Motivation

**Figure 2:**
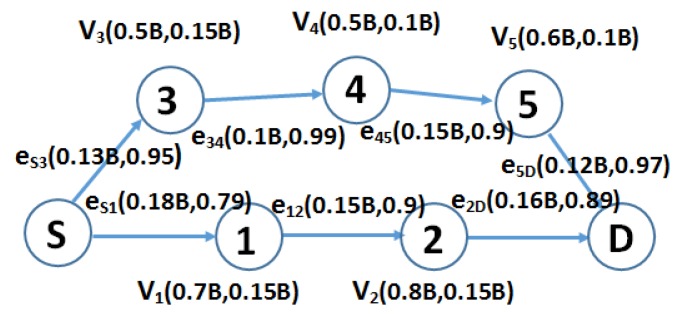
The sample network illustrates the benefit of a stability-aware routing scheme for minimizing energy consumption and reliable data transmission in energy harvesting wireless sensor networks (EH-WSNs).

Motivated by the research mentioned in Section 2.1, this paper proposes a stability-aware routing method for reliable data transmissions on EH-WSNs, named stability-aware geographic routing in SAGREH, to show that performance improvements for EH-WSNs can be achieved by incorporating residual energy, energy harvesting, link quality, and location information in the routing decision. The motivation of the proposed protocol is illustrated by the network scenario shown in Figure 2. In this figure, there are two available routes between a source, S, and a destination, D, or $p_{1}$ and $p_{2}$, which are represented as (S, 1, 2, D) and (S, 3, 4, 5, D), respectively. Here, B is the maximum battery of each node $v_{i}$. In $e_{ij}\left(z,w\right)$, *z* indicates the amount of energy consumption, and *w* indicates estimated packet reception rates. The relationship between the estimated packet reception rate and the number of retransmissions was compared to the study by Kuruvila *et al.* [24], who considered routing with acknowledgment. Specifically, a packet is retransmitted between two nodes until it is received and acknowledged correctly. They showed that the best value for the number of packet retransmissions, *u*, was not a constant and could be solved by:

(1) u×PRR=1

If $p_{1}$ is chosen, the total hop count on this path is four, and the estimated PRR on route $p_{1}$ is PRR($p_{1}$) = 0.95×0.99×0.9×0.97 = 0.821. If $p_{2}$ is chosen, the total hop count on this path is three, and the estimated PRR on route $p_{2}$ is $PRR(p_{2})=0.79\times 0.9\times 0.89=0.633$. Moreover, the average energy consumption of $p_{1}$ and $p_{2}$ is equal to 0.51 B × *u* or 0.49 B × *u*, respectively, and the residual energy of the network after transmitting one packet over $p_{1}$ or $p_{2}$ is 3.75 B − 0.51 B/0.821 = 3.141 B or 3.75 B − 0.49 B/0.633 = 2.98 B, respectively; 3.75 B is the total residual energy and energy harvesting at each node in the network. The reliable routing path from source to destination is $p_{1}$ because its average consumed energy is lower and it provides a higher estimated packet reception rate, making it the best choice for a network. If only residual energy, consumed energy, and harvested energy nodes on the path are considered, then $p_{2}$ should be chosen. However, compared with $p_{1}$, $p_{2}$ is an unstable path because the packet reception rate on this path is lower, so the total energy needed to transmit a packet successfully is higher.

The objective of the proposed SAGREH approach is to select the route that not only has minimum energy consumption and maximum residual energy, but also gives high estimated packet reception rate to provide a reliable route. Because the performance of a network depends heavily on the PRR, the PRR is used as an input metric to select a stable routing path.

## 3. System Model

We considered EH-WSNs consisting of N energy harvesting sensors nodes randomly distributed in a two-dimensional area a × a. Each sensor node is equipped with omnidirectional antennas and has a certain radio transmission range R. The energy model and wireless link model used in this paper are presented in Section 3.1 and Section 3.2, respectively.

A successful packet reception between two nodes, $v_{i}$ and $v_{j}$, is influenced by wireless channel conditions and channel conflict and is modeled on a PRR.

### 3.1. Energy Model

The energy model of EH-WSNs in this paper consists of three components, *i.e.*, harvested energy, packet energy, and node’s residual energy, which will be presented in detail as follows.

(a) **Harvested energy** ($E_{h}$) is the amount of energy harvested from the environment at each node during a unit time to maintain the operation of the sensor node (e.g., sensing, transmitting, and receiving packets). Even when sensor nodes are located where it is difficult to recharge or replace a battery, they should harvest energy from the environment or other energy sources, such as vibration, thermoelectricity, or acoustic noise, to prolong their lifetime.

In this paper, sensor nodes that harvest energy through solar cells were chosen. The amount of harvested energy at each node depends on light intensity. Because of the amount of harvested energy at each node is different, the effect of random light intensity in the environment can be expressed as

(2) $$E_{h}=\beta_{i}\times E_{max}$$

where $E_{max}$ is the harvested energy corresponding to the highest light intensity, $\beta_{i}$ represents a multiplication coefficient which is uniformly distributed in [0, 1].

(b) **Packet energy** ($E_{e}$) is the amount of energy consumed by a node when sensing, receiving, or transmitting a packet with a packet length of *L* (bits), and the amount of energy consumed is denoted as $E_{se}$, $E_{rx}$, and $E_{tx}$, respectively. The wireless propagation channel is affected by the large-scale path loss model [25]. Thus, $E_{se}$, $E_{rx}$, and $E_{tx}$ are expressed as

(3) $$E_{se}=\alpha_{1}\times L$$

(4) $$E_{rx}=\alpha_{2}\times L$$

(5) $$E_{tx}=\left(\alpha_{3}+\alpha_{4}d^{\beta }\right)\times L$$

where *d* (d≤R) is the distance between transmitter and receiver, *β* is the path loss exponent, $\alpha_{1}$ = 50 nJ/bit, $\alpha_{2}$ = 135 nJ/bit, $\alpha_{3}$ = 45 nJ/bit, $\alpha_{4}$ = 10 pJ/bit/m^2^ and 0.001 pJ/bit/m^4^ when *n* = 2 and 4, respectively [26].

(c) **Residual energy at node** $v_{i}$ ($RE_{i}$) is a sink node supplied by unconstrained energy with the residual energy of other nodes considered. The residual energy of node $v_{i}$, ($RE_{i}$), after transmitting through path $p_{k}$, can be expressed as:

(6) $$RE_{i}\left(p_{k}\right)=B\;-\rho \times E_{e}+\beta_{i}E_{max}$$

where *ρ* represents the number of packets transmitted from node $v_{i}$.

A specific sensor node can communicate with others if the residual energy of node $v_{i}$ is greater or equal to a packet energy, *i.e.*, $RE_{i}\geq E_{e}$.

### 3.2. Wireless Link Model

In this paper, the received signal power at distance *d* from the transmitter can be expressed by [25,27]:

(7) $$P_{r}=\frac{P_{t}G_{t}G_{r}\lambda^{2}}{{\left(4\pi \right)}^{2}d_{o}^{2}L}\times (\frac{d_{o}}{d}{)}^{\beta }\times 10^{0.1X_{\sigma }}$$

where $P_{t}$ is the transmitted signal power, $G_{t}$ and $G_{r}$ are the antenna gains of the transmitter and the receiver, respectively, L(L≥1) is the system loss, and *λ* is the wavelength, the mean of $X_{\sigma }$ is zero, reference distance $d_{0}$ is 1 m, *β* is the path loss exponent with fixed value between 2 and 6.

A packet can be successfully received at the receiver if the received power is greater than or equal to a threshold. The probability of this event happening is denoted as $p_{S}\left(d\right)$. Therefore, we have

(8) $$p_{S}\left(d\right)=P\left(P_{r}\geq P_{th}\right)=\left(\frac{P_{t}G_{t}G_{r}\lambda^{2}}{{\left(4\pi \right)}^{2}d_{0}^{2}L}\times {\left(\frac{d_{0}}{d}\right)}^{\beta }\times 10^{0.1X_{\sigma }}\right)\geq P_{th}$$

**Figure 3:**
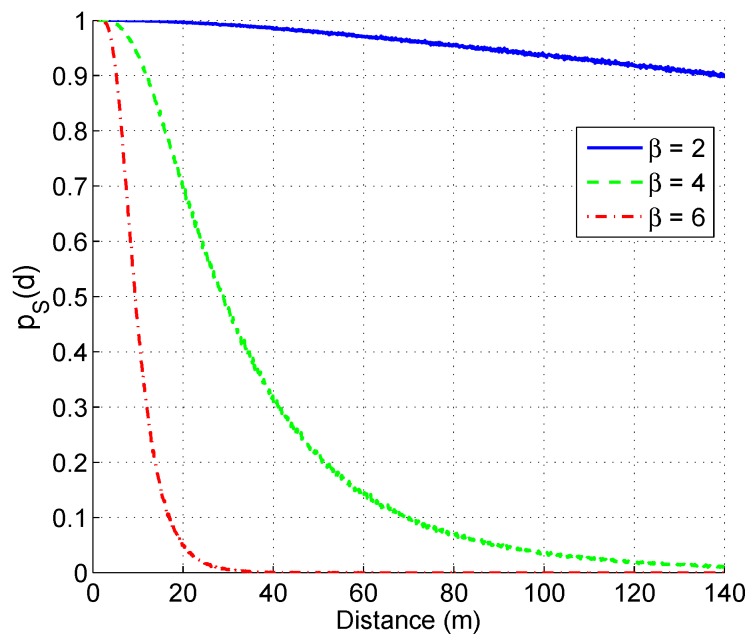
Illustration of the relationship between distance and $p_{S}\left(d\right)$ between two nodes with β=2, β=4, and β=6.

Figure 3 presents $p_{S}\left(d\right)$ as a function of the distances from a transmitter to receivers with different *β*, $P_{t}$ = 1 mW, *f* = 914 Mhz, $P_{th}=10^{-12}$ W, the mean of $X_{\sigma }$ is zero, standard deviation *σ* is 12. As we can see in Figure 3, when the receiver is far from the transmitter, the $p_{S}\left(d\right)$ is lower, and *vice versa*. It should also be noticed that $p_{S}\left(d\right)$ is not linearly proportional to the distance *d*.

With the given above, the probability $p_{S}\left(d\right)$ that a node at distance *d* to the transmitter can successfully receive a packet from the transmitter, the number of receivers that successfully receive a packet within the distance *R* of the transmitter is calculated as

(9) $$N_{tx}\left(R\right)=\int_{0}^{R}2\pi d\eta p_{S}\left(d\right)dd$$

where $\eta =\left(N-1\right)/a^{2}$ is the density of receivers, *N* is the number of sensor nodes in the network, and *a* is the network size.

The average number of receivers within distance *R* of the transmitter is given by

(10) $$N_{n}\left(R\right)=\eta \pi R^{2}$$

As an important reliability index of communication among sensor nodes, packet reception rate (PRR), is defined as the percentage of nodes that successfully receive a packet from the transmitter among the receivers. Specifically, the PRR is taken as the ratio between the number of nodes within distance *R* of the transmitter that successfully receive packets from the transmitter and the average number of receivers within distance *R* of the transmitter; *i.e.*,

(11) $$PRR\left(R\right)=\frac{N_{tx}\left(R\right)}{N_{n}\left(R\right)}$$

Our proposed SAGREH uses the above PRR as a metric to select the most stable multi-hop routing path. The details of route selection will be presented in Section 4.2.

## 4. Stability-Aware Geographic Routing in EH-WSNs (SAGREH)

### 4.1. Energy Harvesting Wastage-Aware Protocol (EHWA)

Despite significant development in WSNs, battery capacity remains a major limitation to WSN applications. In former energy-aware routing protocols, sensors were powered by batteries with limited capacities, and routing decisions were made based on the energy consumption of receiving or transmitting packets or residual energy at each node. The objective of these protocols was to maximize network lifetime, which measures the amount of time elapsed before the first sensor (or a fraction of sensors) is dead. Even with state-of-the-art energy harvesting technologies, the rate of harvested energy may be lower than the typical power consumption level. Consequently, the recent energy-harvesting routing algorithms still focus on the network lifetime problem. This study used the EHWA to represent energy-harvesting routes to maximize network lifetime. Specifically, the EHWA selected the routing path that results in the maximum total residual energy network energy after routing realization, which is expressed as [28]

(12) $$\begin{array}{c} \underset{p_{k}}{\mathrm{arg max}}\;\left\{\sum_{\forall i}RE(p_{k})\right\}\\ s.t.\;p_{k}\in P\\ \;\;\;i\in N \end{array}$$

where *P* is a set of all available routing paths $p_{k}$, *i* represents node $v_{i}$, which belongs to path $p_{k}$.

The EHWA tends to maximize the total residual energy, or the total consumption along the route needs to be minimized, which is intuitive. Moreover, authors propose a route selection scheme that takes into account the predicted wastage energy due to overcharge of finite battery. However, they do not consider the influence of link quality on the network performance. To solve this problem, we propose a SAGREH routing protocol which is aware of the reliability of packet sending from source to sink by selecting the most stable path among available ones.

### 4.2. SAGREH Protocol

In the paper [29], a light-weight opportunistic forwarding (LWOF) scheme and an energy efficient MAC protocol (*i.e.*, LWMAC) with optimized preamble’s length used in LWOF are proposed to reduce energy consumption in wireless sensor networks. As mentioned in [11], a forwarding node is selected based on the location-address semantic (*i.e.*, greedy forwarding) and this location address semantic is valid in many sensor networks because sensor data are normally tagged with the location information. In our paper, we also employ greedy forwarding in selecting forwarding nodes. It should be noticed that the greedy forwarding algorithm with 60° sector used in [29] is for eliminating hidden forwarder. Moreover, according to the opportunistic forwarding scheme, it is required that only one forwarding node is selected among group of nodes and the forwarding node selection process gradually builds a single path from source node to sink node to deliver the data packet during the data packet transmission phase. However, in our paper, the proposed SAGREH exploits greedy forwarding to limit the forwarding area of the control packet (*i.e.*, Route Request (RREQ) packet) in the route exploration phase, not data transmission. The RREQ packets reach the sink node through many paths. Then, among these paths, the sink node will select the most stable path specified by Equation (15). The details of path selection will be described as follows.

In a multi-hop case, when a source wants to transmit data to sink, if it chooses a nearer neighbor, the PRR is higher, but the number of hop counts to reach the sink is larger, and *vice versa*. Specifically, for our model, we ask the question: How to perform a trade off between PRR and the number of hop counts in multi-hop routing? We will propose the SAGREH to solve this problem.

The localized routing algorithm for EH-WSNs proposed here has nodes that do not require complete network topology information to perform routing tasks. It assumes that each node knows the location information for itself and the sink node. Routing paths are dynamically constructed during the packet forwarding phase, reducing the cost of control message overhead. All nodes transmit with equal transmission power, thus, the nodes have fixed and equal transmission ranges, or *R*.

Most geographic routing finds greedy forwarding impossible; (*i.e.*, when approaching a hole with no relay node, routing reverts to the perimeter mode). As shown in Figure 1, when the localminimumproblem occurs at node A, the node will seek other routing paths to sink around the void area; if a routing path to the sink area exists from node A, it does not include the node located within the void area. This is called the perimetermode. SAGREH takes into account the residual energy, energy harvesting, location, and link quality information of neighboring nodes to select the routing path. In the following section, the routing path from the source to the sink node is described.

**Figure 4:**
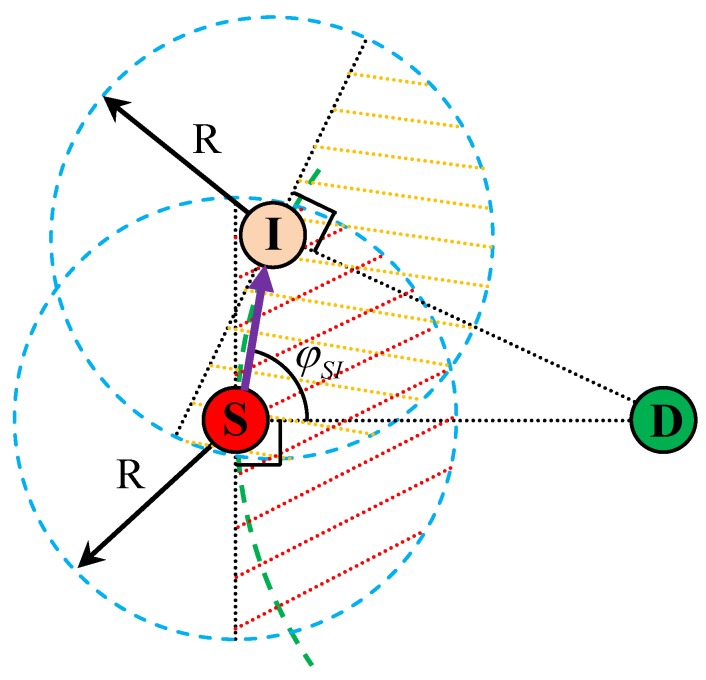
An example scenario of the selection of a neighbor node based on the residual battery, energy harvesting, and location information in the proposed stability-aware geographic routing in energy-harvesting wireless sensor network (SAGREH) routing protocol.

Figure 4 shows the influence of residual energy and location information on the process of forwarding node selection in SAGREH. The circle with radius R around the nodes indicates the transmission range. The distance between source node (S) and sink node (D) is $d_{SD}$. If $d_{SD}$ is greater than the transmission range R, intermediate nodes are needed to forward the data packet from S to D. The detailed process of determining forwarding nodes in our proposed SAGREH is described as follows.

- **Step 1 (determining the neighbor nodes):** Firstly, the source node includes its location in a RREQ packet and broadcasts this packet. An intermediate node who receives this RREQ packet can retrieve the location of source node, then it uses this location information, its own location, and residual energy information to evaluate whether it is allowed to forward to RREQ. Specifically, an intermediate node is only allowed to forward the RREQ packet if:
  (i) The distance from it to previous hop is less than transmission range.
  (ii) To prevent backward packet transmission, the selected forwarding node must locate in a half circular are toward the sink node. To determine a node in the half circle, the angle $\varphi_{SI}$ between the two lines, SI and SD, is calculated. Based on the law of cosines, angle $\varphi_{SI}$ can be calculated as follows
  (13) $$\varphi_{SI}=ar\cos\left(\frac{d_{SI}^{2}+d_{SD}^{2}-d_{ID}^{2}}{2d_{SI}d_{SD}}\right)$$
  where $d_{SI}$ and $d_{ID}$ are the distance between nodes S and I, and the distance between nodes I and D, respectively. If $\varphi_{SI}$ is less than or equal to 90°, node I locates in the half circle toward node D.
  (iii) Its residual energy is greater than or equal to its packet energy $E_{e}$.
  These conditions are summarized in Equation (14) as
  (14) $$\left\{\begin{array}{c} d_{SI}\leq \;R\\ \varphi_{SI}\leq \;90{}^{\circ}\\ RE_{I}\leq \;E_{e} \end{array}\right.$$
- **Step 2:** The above forwarding node selection process in the route exploring phase is repeated until node D is reached.

Aside from using the residual energy and location information for path selection, the estimated PRR of the wireless link is also employed to select the most stable path from all available paths. The detailed process of stable path selection is presented as follows.

- **Step 1:** Initially, source node (S) broadcasts its RREQ packet to its neighbors. The RREQ consists of its node ID and location, the Path PRR field which is initially set to 1, sequence number to prevent from routing loop, and Time to Live (TTL) to avoid RREQ from flooding throughout the network.
- **Step 2:** A sensor node which receives the RREQ packet from previous node will store previous node’s ID in its Route Cache, uses the previous node’s location and its current location to calculate the PRR of the link between it and previous node by Equation (11) in Section 3.2, then updates Path PRR field in RREQ by multiplying this value by the previous one. Moreover, this sensor node will decrease TTL by one before broadcasting to its neighbors.
- **Step 3:** The above step is repeated until the RREQ reaches sink node (D). After knowing the PRRs of paths, the sink node will select the path whose PRR is the highest; *i.e.*,
  (15) $$\underset{i,j\in p_{k}}{\mathrm{arg max}(\prod PRR_{ij})}$$
  where *i*, and *j* represent node $v_{i}$ and node $v_{j}$, respectively.
  After selecting the path whose PRR is the highest, the sink node adds this path information in the Route Reply (RREP) sending back to source node.
- **Step 4:** After source node receives RREP from sink node, it will forward data packets through this stable path.

## 5. Performance Evaluations

### 5.1. Simulation Environment and Performance Metrics

We use a MATLAB simulator to compare the performance of SAGREH with that of EHWA. The following performance metrics will be used:

- **Packet delivery ratio** (PDR) is defined as
  (16) $$PDR=\sum_{1}^{\Omega }\frac{\#\;packet\;received\;at\;sink}{\#\;packet\;transmitted\;from\;source}$$
  where Ω is the number of topologies.
  In this paper, the PDR of the EHWA protocol-based EH-WSNs and SAGREH were comparatively studied as a function of the radio transmission range and number of nodes.
- **Average consumption energy** ($\bar{CE_{i}}$) is defined as the average energy consumed to transmit a packet from the source node to the sink node and is given by
  (17) $$\bar{CE_{i}}=\frac{1}{\Omega }\sum_{i=1}^{\Omega }CE_{i}$$
  where $CE_{i}$ is the energy consumed over a route to transmit a data packet from a source node to a sink node in the $i_{th}$ network topology, and Ω is total number of network topologies.
- **Average hop count of successful routing path from source to sink** ($\bar{HC_{i}}$) is the average number of hops over a path between a source node and a sink node, as follows
  (18) $$\bar{HC_{i}}=\frac{1}{\Omega^{\prime }}\sum_{i=1}^{\Omega^{\prime }}HC_{i}$$
  where $HC_{i}$ is the number of hops of a successful routing path from a source node to a sink node in the $i_{th}$ network topology, and $\Omega^{\prime }$ is the total number of topologies with a successful routing path from the source to the sink, $\Omega^{\prime }$ ∈ Ω.

Network parameters used in the simulations are presented in Table 1. For this evaluation, it was assumed that all the packets were available in the buffer.

In all scenarios, the sink node location was the same, while the source node was randomly located in the network area. The mobility of the source node and intermediate nodes was represented by different random locations in each network topology.

**Figure 5:**
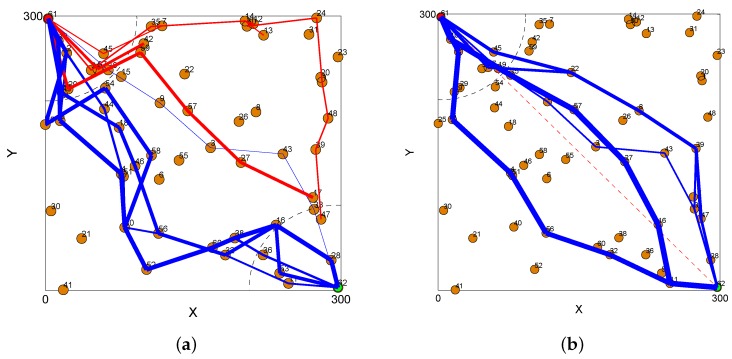
Accumulated routing path corresponding to (**a**) EHWA and (**b**) SAGREH after transmitting 15 packets.

Figure 5a,b show the accumulated routing paths from a source to a sink node, which were obtained after transmitting 15 packets using the EHWA and SAGREH. The data packets that traveled over the edges are represented by the thickness of the corresponding line. In Figure 5a, some disconnect is evident in the path, indicating that some packets could not reach the sink node. However, the routing path between the source and sink nodes can be established. Figure 5b presents an evaluation of the performance of the proposed SAGREH protocol.

### 5.2. The Impact of Radio Range on PDR, Hop Count, and Energy Consumption between EHWA and SAGREH

**Figure 6:**
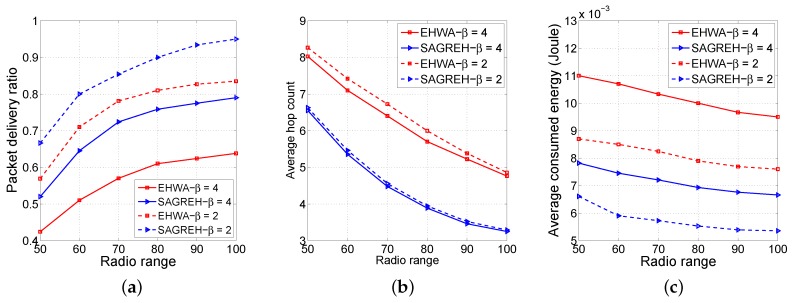
(**a**) The Packet delivery ratio (PDR), (**b**) average hop count, and (**c**) average energy consumption as functions of radio range.

Figure 6 shows the PDR, average hop count, and average energy consumption as functions of the radio range with 100 nodes. Radio range varied from 50 m to 100 m.

From Figure 6a, radio range influences the packet delivery ratios for both the EHWA and SAGREH. As the nodes’ radio range increases, the number of neighboring nodes increases, and the possibility that data packets traveled through a shorter route path was higher, which increases the reliability of the path between a source node and a sink node. SAGREH provides a significantly higher PDR than the EHWA when the path loss exponent, represented as *β*, degrades from 4 to 2 because the PDR closely depends on the PRR of each route path. Based on Equation (15), SAGREH tends to select the path that supports the highest PRR, while EHWA tends to selects the routes that maximize the residual energy of the network. Therefore, SAGREH provides a path having higher PRR than EHWA, leading to the EHWA providing a significantly lower PDR than SAGREH.

As shown in Figure 6b, in regards to the average number of hop counts, the higher the radio range is, the lower the average obtained hop count. This is because a node can have more neighbors if it has a longer transmission range. When the radio range increases, SAGREH improves the average hop count compared to the EHWA; for example, with a *β* = 2, SAGREH improves the count by 20% compared to EHWA’s average hop count at the 50 m radio range, and it improves by 32% at the 100 m radio range because in a small radio range (*i.e.*, 50 m), a specific node does not have many neighbors to select from. This leads to less hop count variances for the paths selected by the EHWA and SAGREH and can explain why EHWA’s and SAGREG’s PDRs were proportional to the radio range, as shown in Figure 6a. The difference is proportional because the hop count was inversely proportional to the PRR of the route path. We can see that the EHWA tends to select longer routes formed by nodes with the highest residual energy, and the SAGREH selects a shorter route formed by nodes that not only satisfied the energy condition and PRR condition but were also closer to the sink node.

From Figure 6c, decreasing the average hop count leads to decreased average energy consumption in each route. The higher $\bar{HC_{i}}$ in the EHWA led to higher $\bar{CE_{i}}$. The degree of change in the average energy consumption of the EHWA was larger than that of SAGREH when the *β* degraded from 4 to 2 because the average energy consumption depends on the hop count and PDR. Moreover, the degree of changes to the PDR of the EHWA was much larger than that of the SAGREH, as the *β* decreased from 4 to 2, and the degree of changes in average hop count for the SAGREH was lower than that for the EHWA. Therefore, the SAGREH provides better average consumption energy than the EHWA.

### 5.3. The Impact of Number of Nodes on PDR, Hop Count, and Energy Consumption between EHWA and SAGREH

**Figure 7:**
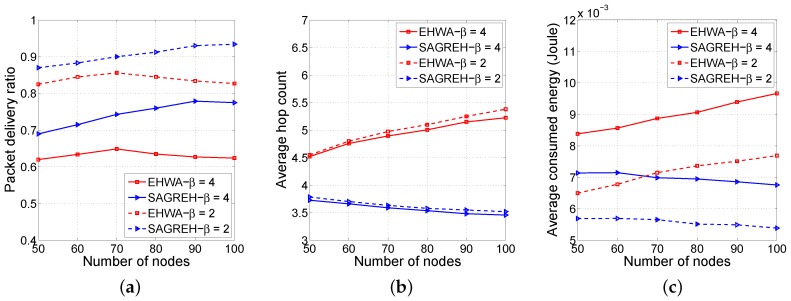
(**a**) The PDR, (**b**) average hop count, and (**c**) average energy consumption as a functions of the number of nodes.

Figure 7 shows the performance of both the EHWA and SAGREH in terms of the PDR, average hop count, and average energy consumption as functions of the radio range, which was 90 m. The number of nodes varied from 50 to 100.

Moreover, SAGREH provides better PDR compared to EHWA. When the number of nodes increased, the PDR of SAGREH increased. While the PDR of the EHWA slightly decreased because the PRR of a route heavily depends on the hop count.

Figure 7b shows an interesting result; when the number of nodes increased, the $\bar{HC_{i}}$ of the EHWA also increased, but the $\bar{HC_{i}}$ of SAGREH slightly decreased because the EHWA tended to select longer routes to avoid nodes with low energy levels. In the worse cases, it even selected spiral routes. The SAGREH selected shorter routes to maximize the PRR of routing path from source node to sink node. With a given radio range, the SAGREH provided more stability for routing than EHWA did. Specifically, the average hop count of the SAGREH did not depend on the number of nodes. For instance, with a *β* = 4 and the number of nodes varying from 50 to 100, the average hop count varied from 3.73 to 3.46, while the average hop count of EHWA changed significantly according to the number of nodes. For example, with a *β* = 4 and the number of nodes changing from 50 to 100, the average hop count change was from 4.52 to 5.23.

Because the EHWA selected longer paths with a higher probability of packet loss, it consumed much more energy than the SAGREH protocol. Figure 7c shows that the $\bar{CE_{i}}$ of SAGREH was unchanged, while the $\bar{CE_{i}}$ of the EHWA increased. This shows the remarkable advantages of the proposed protocol compared to the EHWA in terms of energy consumption.

## 6. Conclusions

This research proposed stability-aware geographic routing in EH-WSNs, which considered not only residual energy and harvested energy, but also estimated PRR of wireless link and location information to select routing paths from a source node to a sink node.

The simulation results showed that the SAGREH outperformed the EHWA in relation to PDR, average hop count, and average energy consumption. Specifically, the SAGREH provided a higher PDR and a lower average hop count and energy consumption than the EHWA did, meaning that the proposed method provided better performance in a more realistic environment.

Future research will investigate stability-aware energy harvesting for reliable high-speed data transmission in multi-rate and realistic physical layers. 

## Figures and Tables

**Table 1:** Network parameters.

Network Parameters	Value
Simulator	MATLAB R2014a
Simulator time	500 s
Network area	500 m × 500 m
Radio transmission range	50–100 m
Number of nodes	50–120
MAC protocol	IEEE 802.11
Data rate	20,000 bps
Number of topologies	10,000
Packet size	512 bytes
Maximum harvested energy $E_{max}$	25 × $10^{-6}$ J/s
